# Exploring optimal control of epidemic spread using reinforcement learning

**DOI:** 10.1038/s41598-020-79147-8

**Published:** 2020-12-16

**Authors:** Abu Quwsar Ohi, M. F. Mridha, Muhammad Mostafa Monowar, Md. Abdul Hamid

**Affiliations:** 1 Department of Computer Science and Engineering, Bangladesh University of Business and Technology, Dhaka, Bangladesh; 2 Department of Information Technology, Faculty of Computing and Information Technology, King Abdulaziz University, Jidda, 21589 Kingdom of Saudi Arabia

**Keywords:** Data mining, Machine learning, Statistical methods

## Abstract

Pandemic defines the global outbreak of a disease having a high transmission rate. The impact of a pandemic situation can be lessened by restricting the movement of the mass. However, one of its concomitant circumstances is an economic crisis. In this article, we demonstrate what actions an agent (trained using reinforcement learning) may take in different possible scenarios of a pandemic depending on the spread of disease and economic factors. To train the agent, we design a virtual pandemic scenario closely related to the present COVID-19 crisis. Then, we apply reinforcement learning, a branch of artificial intelligence, that deals with how an individual (human/machine) should interact on an environment (real/virtual) to achieve the cherished goal. Finally, we demonstrate what optimal actions the agent perform to reduce the spread of disease while considering the economic factors. In our experiment, we let the agent find an optimal solution without providing any prior knowledge. After training, we observed that the agent places a long length lockdown to reduce the first surge of a disease. Furthermore, the agent places a combination of cyclic lockdowns and short length lockdowns to halt the resurgence of the disease. Analyzing the agent’s performed actions, we discover that the agent decides movement restrictions not only based on the number of the infectious population but also considering the reproduction rate of the disease. The estimation and policy of the agent may improve the human-strategy of placing lockdown so that an economic crisis may be avoided while mitigating an infectious disease.

## Introduction

Through a pandemic situation, the foremost intention is to produce a vaccine that provides immunity over a particular infectious disease. However, an effective vaccine may take years to develop depending on the disease and some certain criteria. While investigating the vaccine, the loss of a pandemic is to be controlled via proper clinical support and by reducing the expanse of the disease. Nevertheless, assuring proper clinical care is not possible in a pandemic situation due to a large number of infections over the available limited clinical support. Therefore, lessening the expanse of a disease is the first and foremost effort to overcome the devastation of a pandemic disaster.

Pandemics are often caused by diseases that transmit through person-to-person close contact^1^. At present, pandemics are caused by flu such as Swine flu^2^, and Coronavirus^3,4^. Different intervention means are proven to reduce the devastation of a pandemic outbreak^5^. However, these interventions often cause an economic breakdown, and it is not possible to reduce the impact of a pandemic without it^6^. Therefore, a pandemic situation raises challenges to balance the viral spread and a steady economy.

Due to the current COVID-19 pandemic, researchers have been investigating various strategies to reduce the pandemic’s desolation while striving economic balance. Through several research endeavors, various lockdown strategies have been proposed, such as age-based lockdown^7^, n-work-m-lockdown^8^, and so on. However, age-based lockdown should not apply for a disease that is critical for all ages. Also, repeated n-work-m-lockdown (n days without lockdown followed by m days of lockdown) strategies may not ameliorate critical pandemic situations. The current challenge of a pandemic situation raises cases such as, (a) is placing a long time lockdown the only way to mitigate a pandemic?, (b) should we place lockdowns while the pandemic situation does not ameliorate?, (c) how should the resurgence of the pandemic be handled?, (d) while mitigating a pandemic, how we could also balance the economical circumstances? In our research endeavor, we attempt to resolve these concerns by combining reinforcement learning and virtual environment based epidemic analyses.

In aspects of mathematics and computer science, the challenge of maximizing a constraint (the economic balance) while minimizing some other factor (reducing the spread of disease) is referred to as an optimization problem. The knowledge of making the best decision of an optimization problem is termed as a policy. The best policy may be found using Reinforcement Learning (RL). In RL, a machine is defined as an actor or agent. The actor performs some actions in an environment and earns a reward for every activity. The actor’s goal is to find such a policy that will cause it to acquire the maximum possible reward. An RL agent can adapt actions like animals through proper setup, even like the intelligent ones^9^.

Previously, the field of RL was enclosed with implementing dynamic programmings with tabular functions. Q-Learning^10^, Double-Q Learning^11^ were the fundamental methods of RL. However, the vast improvement of Deep Learning (DL) has enabled it to use RL strategies^12^. In recent times, instead of using tabular functions, Deep Neural Networks (DNNs) are implemented in RL^12^. Deep Reinforcement Learning (DRL) has improved the previous fundamental methods to be implemented using Deep Q-Learning, Double Deep Q-Learning (DDQN), and so on. Also, the current improvement of RL has attracted researchers and therefore, various new implementations are currently available.

The present state of DRL has proven its strength in various platforms such as playing Atari like human^13^, chatting like human^14^, playing hide and seek^15^, and so on. Furthermore, recent improvements in DRL have resulted in beating humans in poker^16^, go^17^, and even in DOTA-2^18^. DRL is astonishing humans by generating new optimal ideas that were never thought of.

Being inspired by the recent improvements of DRL, in this paper, we search for some optimal ideas on pandemic mitigation. To carry out the exploration, we implement a virtual environment that simulates a pandemic crisis. We consider the disease that causes the pandemic to be transmitted in close contact. A short term memory based DDQN is used as an RL agent. The agent’s goal is to formulate an optimal strategy so that a pandemic crisis may be mitigated while maintaining economic balance. The contribution of our research endeavor includes:

1. We implement a virtual environment that simulates a pandemic situation and also considers economic circumstances.
2. We illustrate the consequences of placing no lockdown, maintaining social distancing, and placing lockdown. The consequences are derived based on the death of population and economic situations.
3. We investigate optimal strategies to reduce the spread of disease using reinforcement learning. Furthermore, we perform extensive analysis and present the reasoning behind the action.

The rest of the paper is organized as follows: in “Methods”, the mechanism of the virtual environment is disclosed, and the neural network architecture of the agent is defined. In “Results”, we evaluate the virtual environment and investigate to discover an optimal agent. Then, we explore various control sequences to reduce the disease’s spread and consume our effort to find and analyze the optimal control sequence generated by the agent. Finally, “Discussion” concludes the paper.

**Figure 1:**
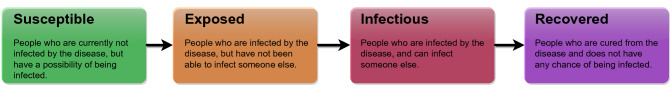
The diagram illustrates the different stages of an SEIR compartmental model. Although it can be observed that the infectious population further approaches to recovered state, a portion of the infectious population may not survive the disease and lose their lives.

**Figure 2:**
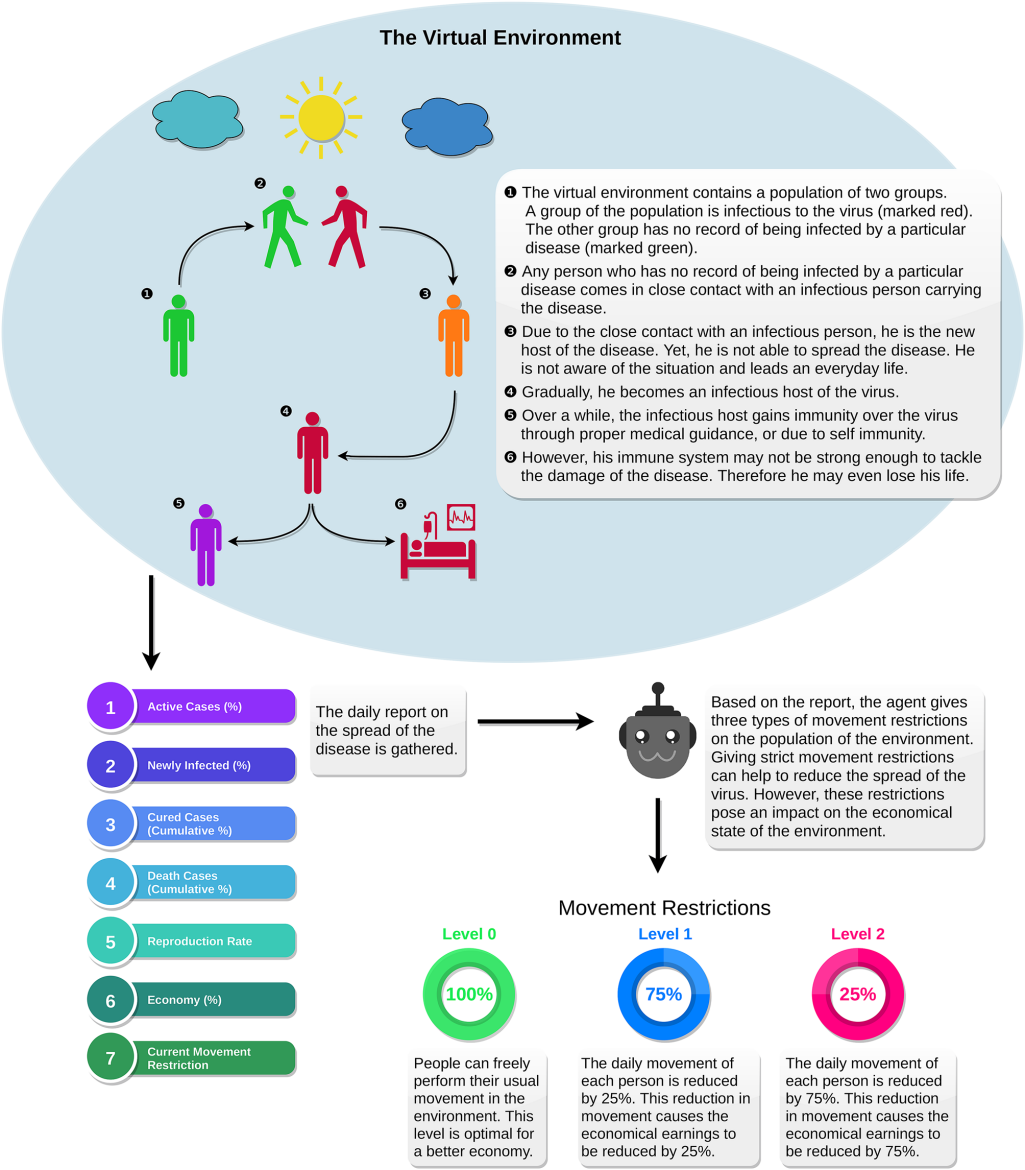
The infographic illustrates the overall dynamics of the virtual environment, environment features, and the agent’s possible actions. Notably, level-1 movement restriction is similar to maintaining social-distancing, and level-2 movement restriction is similar to placing a nationwide lockdown.

## Methods

To study epidemiology, various compartmental models are being implemented^19^. Compartmental models define a simple mathematical foundation that projects the spread of infectious disease. Furthermore, different mathematical models are being presented to illustrate the relationship of population heterogeneity and the present crisis of pandemic^20^. These compartmental models are mostly generated using ordinary differential equations (ODE)^21^.

Although ODE and other mathematical methods are sufficient in modeling an infectious disease, we argue that they are not suitable for training an RL agent as they lack randomness of being infected, cured, and death. Mathematical models do not include any super-spreaders^22^, and lacks randomness. Randomness is required so that RL agents do not overfit for a certain number of initial parameters and generate more uncertainty. The randomness can be considered as comparable to the data augmentation process in deep learning. Data augmentation often helps DNN models avoid overfitting and help achieve better generalization in unseen data^23^. Moreover, RL environments must be dynamic. General ODE models are static, and transforming them into a dynamic state may require additional parameters^24^ that often becomes complex. Therefore, we avoid implementing traditional ODE models and implement a virtual environment that mimics diseases’ transmission.

The virtual environment is used to generate states and results based on some particular actions. The virtual environment is designed based on the SEIR (Susceptible-Exposed-Infectious-Recovered) compartmental model. Figure 1 depicts the different stages of SEIR compartmental model. Due to the randomness in various transitions, implementing virtual compartmental models make the problem more challenging. The virtual environment is designed in a 2D grid where the population can randomly move. In each day, the population performs a fixed number of random moves. In Fig. 2, an info-graphic representation of the environment and the training process is illustrated. In “Results” section, a broad discussion is presented to substantiate the virtual environment.

### Transmission stages

In the environment, susceptible individuals are infected if they are in close contact with an infectious person. Initially, the infected population is in the exposed stage. After 1–2 days, individuals of the exposed stage is further transmitted to the infectious stage. In this stage, individuals can transmit the disease. The infectious individuals are either recovered after 21–27 days, or they may even lose their lives. The environment is configured so that around 80% of the infected population may survive.

**Figure 3:**
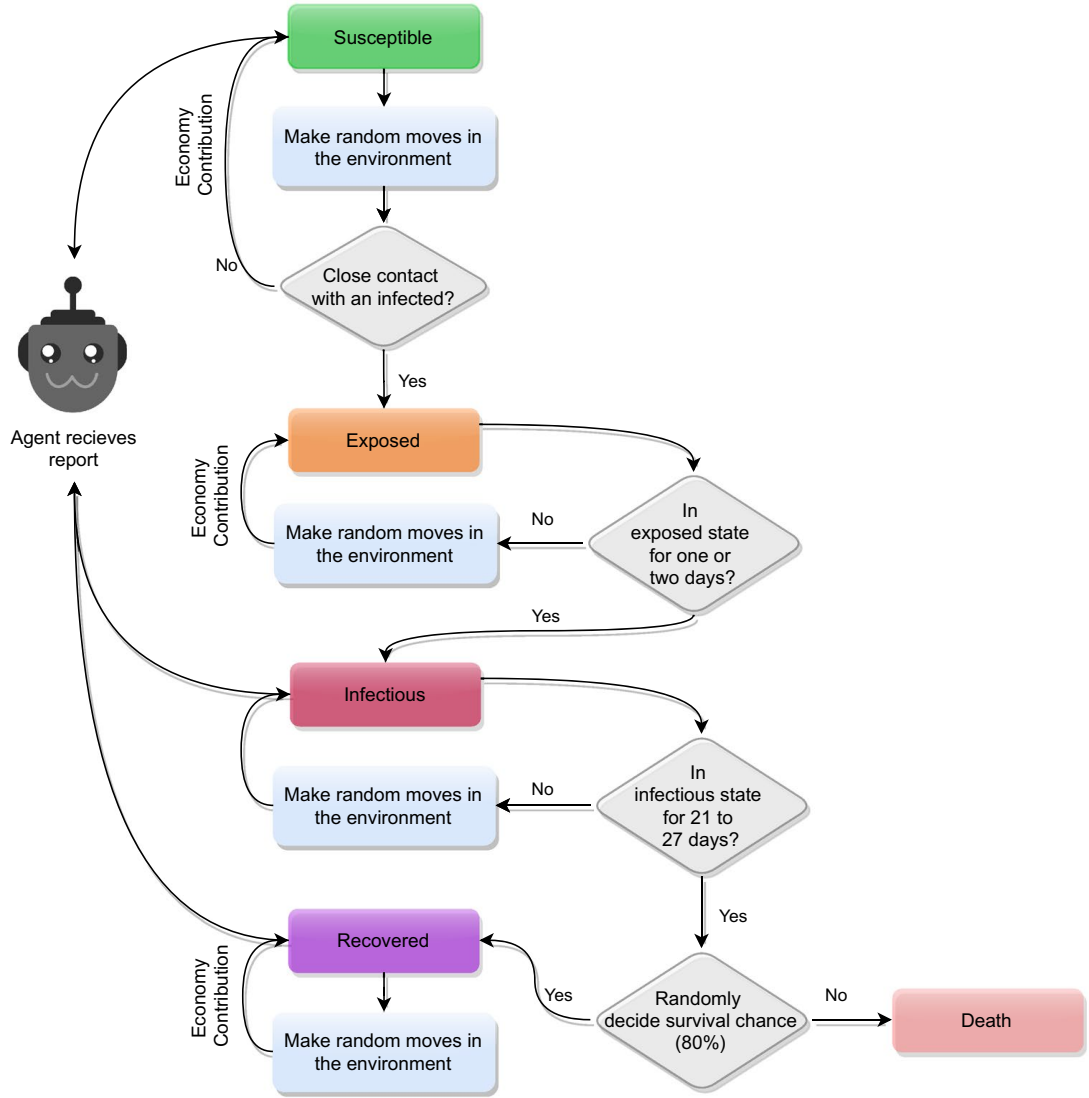
The figure illustrates the algorithmic steps of the environment. The agent receives a daily report of the susceptible, infectious, recovered, and dead population (not presented in the flowchart). Also, the infectious population does not contribute to the economy. The pseudocode of the algorithm is available as a Supplementary file 1.

### Movement restrictions

In the virtual environment, the disease’s spread can be mitigated by reducing the population’s movements. There are three movement restrictions in the environmental setup: level-0, level-1, and level-2. In level-0, no movement restrictions are enforced, and the population makes the maximum movements. In level-1, the movement of the individuals is restricted by 25%. In general, maintaining social distancing and avoiding unnecessary means is equivalent to level-1 restriction^25^. In level-2, the movement is reduced by 75%, similar to a lockdown state^26^. The DRL agent provides these movement restrictions. However, although movement restrictions result in reducing the spread of disease, it causes an economic collapse.

### State genaration

In RL, a state is an observation that passes estimable information to the agent. By analyzing the information, an agent makes an optimal move based on its policy. States can be both finite or infinite. In the virtual environment setup, relevant information about the spread of the disease is passed through a state. Seven parameters are passed as a state of the environment. Figure 2 illustrates the state parameters as infographic. Active cases represent the number of the population who are in the infectious stage. Newly infected refers to the number of the population who have shifted into the infectious stage on a particular day. Cured cases and death cases illustrate the number of people who have been cured and died from the pandemic’s start, respectively. The reproduction rate represents the average number of people who are being infected by the current infectious population. The economy illustrates the daily economic contribution of the population. Along with the states, the current movement restriction is also presented as a state parameter.

### Economical setup

In the virtual environment, each individual contributes to the economy through movement. Therefore, if movement restriction is placed, it has an impact on the economy as well. Each individual contributes a value of [0.8, 1] by moving. People who did not survive can not make any further contributions to the economy and does not exist in the environment. Therefore, the increasing number of death count has also a negative impact on the economy. Also, the infectious population can not contribute to the economy. Therefore, a high number of active cases has also a negative influence on the economy.

### Virtual environment workflow

The environment’s workflow is illustrated in Fig. 3 and the Algorithm contains the pseudocode of the corresponding virtual environment. In the beginning, the virtual environment includes a susceptible and infectious population. Every day, each individual contributes to the economy by making some random movements. Moreover, each individual’s economic contribution is kept a random value in scale [0.8, 1]. A susceptible individual will get into the exposed state if he/she collides with an infectious individual. The agent is not reported any information related to the exposed state. It is theoretically valid, as each individual does not have any health issues in the exposed state. Moreover, there is no scientific process to justify that a person is in an exposed condition. The number of days staying in the exposed state is random per capita, and it is limited to 1–2 days after the collision course. Then the individual enters the infectious form. Persons in exposed and infectious states still perform random movements in the environment. However, a contagious individual can not contribute to the economy. Individuals in the infectious state can be considered patients. Due to the illness, he/she can not work yet still cause contact with other individuals. After 21–27 days, an infectious individual may get recovered. However, roughly 20% of the overall infectious population is dead and removed from the environment. The recovered individuals further make arbitrary movements, contributes to the economy, yet they do not get infected for the second time.

**Figure 4:**
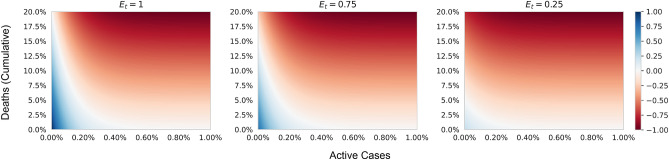
A heatmap representation of the reward function. The horizontal axis represents the percentage of active cases. The vertical axis represents the cumulative death percentage. From left to right, the three heatmaps illustrate the reward distribution in level-0 movement restriction, level-1 movement restriction, and level-2 movement restriction, respectively. In the three restriction levels 0, 1, and 2, the value of $E_t$ is expected to be approximately 1, 0.75, and 0.25, respectively.

### Reward function

In DRL, an action is encouraged and discouraged by a reward function. A reward function encourages an agent to be in a particular state/situation by giving it a high reward for the situation. On the contrary, a specific action or situation is discouraged by giving the agent a low reward. An agent tries to generate such a policy/knowledge so that the agent may avoid the discouraging situation by following the policy. By designing a proper reward function, it is possible to generate such an agent that may follow the human desired situation. For the current environment, the reward function is designed as follows,

1 $$\begin{aligned} \begin{aligned} R(s_t)&= E_t \times e^{-r \times A_t} - s \times D_t \\\\Where,&\\&E_t = \frac{Current\,Economy}{Total\,Population \times M_t} \\&D_t = \frac{Cumulative\,Death}{Total\,Population} \\&A_t = \frac{Active\,Cases}{Total\,Population} \times 100 \\&r = 8 \\&s = 5 \end{aligned} \end{aligned}$$

**Figure 5:**
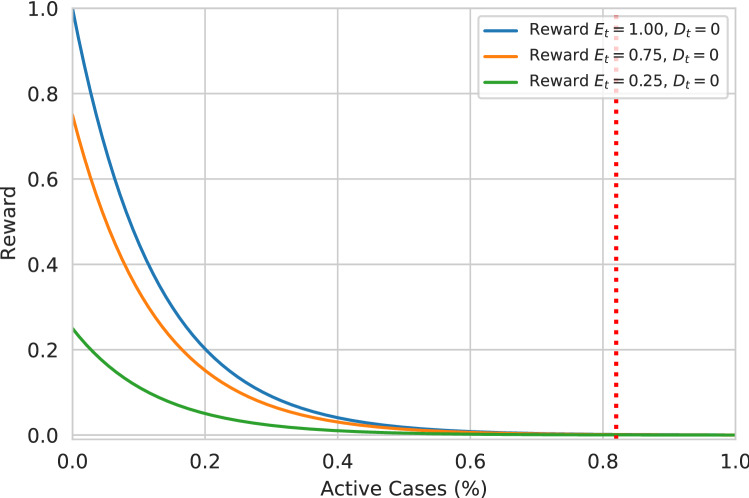
The graph illustrates the decay of reward value concerning the increase in the percentage of active cases (neglecting the cumulative death cases $D_t$ = 0). The value of $E_t$ being 1, 0.75, and 0.25 approximately represents the level-0, level-1, and level-2 movement restrictions. After crossing 0.82% of active cases, the reward of all the different restrictions falls to zero.

The reward function contains three parameters from the environment: the current economy ratio, the current cumulative death ratio, and the current percentage of active cases. Due to the three types of movement restrictions, the economic ratio can be separated into three levels. Due to the direct relationship with movement restriction and economy, level-0, level-1, and level-2 result, the value of $E_t$ is approximately close to 1, 0.75, and 0.25, respectively. However, this can be altered due to high death count and randomness. By avoiding the $D_t$ parameter, the correlation of the economical levels and active cases can be utilized. In Fig. 5, a similar situation is illustrated. By utilizing the graph, it can be perceived that while the active cases are low, the reward prioritizes higher economic stages. The further increase in active cases lessens the reward of higher economic stages. By setting the value of $r=8$, the reward of different economic stages is almost the same (the absolute difference is less than 0.001) after crossing 0.82% active cases. This boundary is thought of as a critical point, after which the economy does not matter. After this boundary, the goal becomes to lessen the surge of the disease. The threshold can be instantiated by the percentage of the population for which proper medical treatment can be guaranteed. Properly selecting the threshold value may reduce death tolls (through adequate medical care) in a real-world scenario. This percentage is often a variable depending on the geographical areas. Furthermore, including the $D_t$ in the reward function, the agent is also encouraged to reduce the death ratio. Figure 4 illustrates the relation of reward function relating to the active case percentage and death ratio in three possible economic stages. The impact of the deaths in the reward function is tuned using the parameter *s*. And $s = 5$ is set to prioritize the negative impact of the deaths.

Both *r* and *s* are the tuning parameters of the reward function. Increasing the value of *r* causes the reward threshold (described in Fig. 5) to be reduced. Whereas, the value of *s* defines the significance of death. A higher value of *s* influences the agent to heavily reduce the death ratio ignoring the economic balance.

**Figure 6:**
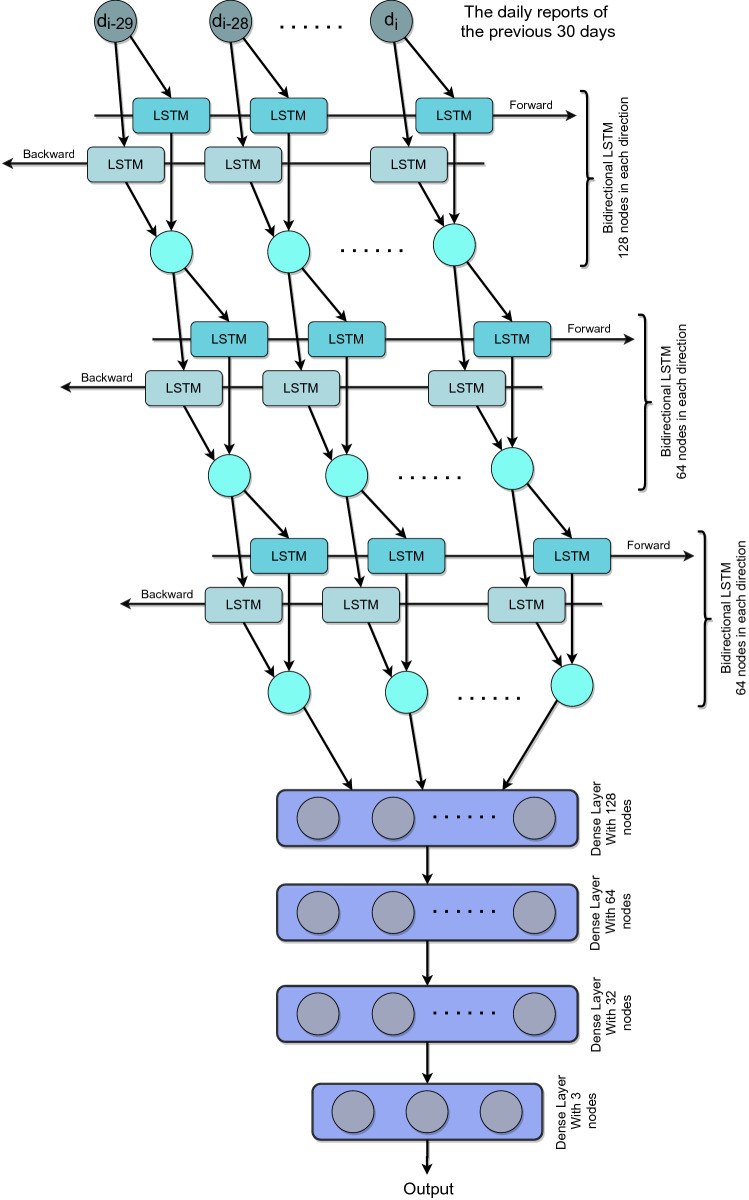
The figure describes the memory-based agent neural network architecture of the agent. The agent uses three bidirectional LSTM layers with 128, 64, and 64 nodes, respectively. It is further followed by four dense layers of 128, 64, 32, and 3 nodes.

### The agent network

The decision process of the DRL can be considered to be a Markov Decision Process (MDP). In MDP, the environment contains a finite set of states *S*, with a finite set of actions *A*. If $s, s^{'} \in S$, and $\alpha \in A$, then the state transition can be represented as,

2 $$\begin{aligned} \tau (s^{'}|s,\alpha ) \end{aligned}$$

The equation states the transition probability of choosing an action $\alpha$, given an environment state *s*, and achieving a new state $s'$. The DRL agent acquires a policy $\pi$ through bootstrapping. Through this policy, the agent performs an optimal action $\alpha _{i}$ for a given state *s*, represented as, $\pi (\alpha _{i}|s)$. The optimal action is chosen based on the state-value function $V^{\pi }(s)$ that defines the chained reward value. The reward value is a chain multiplication of discount value $\gamma$ and state rewards *R*. This can be presented as,

3 $$\begin{aligned} V^{\pi }(s) = {\mathbb {E}}_{\pi } \sum _{k=0}^{n} \left[ \gamma ^{t+k}R_{t+k}|s_0=s \right] \end{aligned}$$

An optimal policy $\pi ^{*}$ finds the best possible state-value function that can be defined as,

4 $$\begin{aligned} V^{*}(s) = max_{\pi } V^{\pi }(S) \quad \forall s \in S \end{aligned}$$

As the transition of an MDP ($\tau (s^{'}|s,\alpha )$) is unknown, a state-action function $Q^{\pi }(s, a)$ is generated. The state action function mimics the value state-value function $V^{\pi }(s)$ and also tries to identify best action $\alpha$. The state-action function greedily chooses the actions for which, it gains the maximum state-value.

The $Q^{\pi }(s, a)$ function is defined as the DRL agent. In the experiment, we study with memory-based DRL agents since the memory-based agent perceives further possibilities and takes optimal decisions and acquires better rewards^27^. We found that the DRL agent makes better actions with a minimal memory of 30 days among different memory sizes. We further investigate to select the optimal memory length in the “Results” section. The agent is implemented using three bidirectional Long Short Term Memory (LSTM). Bidirectional LSTM performs optimally when there exist both forward and backward relationships in a portion of data^28^. In the case of this epidemic data, using bidirectional LSTMs provides the following benefits: (a) select an optimal action based on previous data, and (b) estimate the influence of selecting a particular action. The agent uses three bidirectional LSTM layers, followed by four dense layers. In Fig. 6 the memory-based DRL agent architecture is depicted.

DDQN method is used to train the agent. The DDQN architecture uses an actual agent and a target agent. Traditionally, in DDQN, both agents contain the same network structure. Furthermore, the traditional DDQN training process is implemented to train the architectures^29^. The agent is trained over 7000 episodes and without any pre-knowledge and human interpolation. Random movements are made in the training episodes to explore the environment suitably. The training is started with a random movement ratio of $\epsilon =1$, and it is continuously decayed as $\epsilon = max(\epsilon - \epsilon /(6000), 0.1)$. The discount value ($\gamma$) is set to be 0.9, to propagate the future rewards to any particular state. Mean square error (MSE) is used to calculate the loss between the agent’s predicted and function generated rewards.

**Figure 7:**
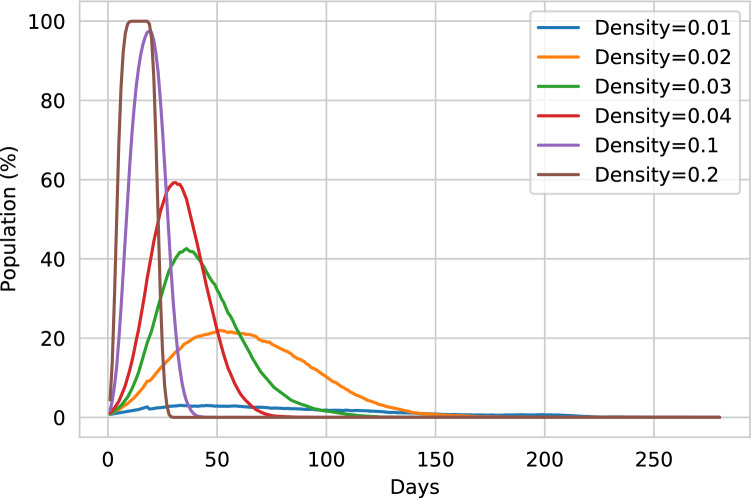
The figure simulates the active cases of the environment, based on different population density. Increasing the density of the population also increases the probability of contact between people. Therefore, the spread of disease also increases.

## Results

The overall implementation is conducted using Python^30^, Keras^31^, and TensorFlow^32^. Matplotlib^33^ is used for graphical representations. The experiments are conducted in a virtual environment implemented on a quadratic time complexity based algorithm, which is provided as a Supplementary file 1. Therefore, we experiment with a limited number of 10,000 population and a default daily movement of 15 steps. In this section, we first evaluate the virtual environment by comparing it with the ODE model. Then we compare the agent’s performance based on different memory lengths and try to resonate an optimal agent. Further, we explore the decisions and exploit the strategy behind the agent’s decision.

### Virtual environment evaluation

Our investigation found that the spread of the disease in the environment acts differently based on the population’s density. In Fig. 7, we illustrate distinguishable waves of active cases over different rates of population density. Due to the high density of the population, the probability of contact between two different person increases. Therefore, the rate of spread of a disease depends on the density of the population. On the contrary, in the environment, a disease’s reproduction rate is not dependent on the population density. In Table 1, the mean and median reproduction rate is reported, tested over different population densities.

**Table 1:** The table compares the reproduction rate in different population density.

Area	Population	Density	$R_{0}$ mean	$R_{0}$ median
1000 $\times$ 1000	10,000	0.01	2.87 ± 0.19	2.84 ± 0.11
708 $\times$ 708	10,000	0.02	3.2 ± 0.30	2.84 ± 0.02
577 $\times$ 577	10,000	0.03	3.4 ± 0.23	2.94 ±0.08
500 $\times$ 500	10,000	0.04	3.4 ± 0.18	2.76 ± 0.11
316 $\times$ 316	10,000	0.1	3.3 ± 0.40	2.73 ± 0.05
224 $\times$ 224	10,000	0.2	3.4 ± 0.12	2.9 ± 0.05

The comparison is represented in a mean±standard-deviation format of the data collected in ten individual runs.

The increase in density does not alter the reproduction rate of the environment. The virtual environment posses nonlinearity in reproduction number. Hu et al. verified that nonlinearity could cause reproductive rates to be at a limit after a particular increase in the density^34^. Yet, the surge of active cases tends to rise while increasing the population density. This scenario can be incarnated by Eq. (6). Higher density causes a higher initial wave of active cases mostly caused by super spreaders. The new infections are caused due to the raised active cases and results in an exponential increase.

The mean and median of the virtual environment’s reproduction rate closely simulates the estimated reproduction rate evaluated in China. To approximate the reproduction rate of COVID-19, Liu et al. evaluated multiple reports from different provinces of China (including Wuhan and Hubei) and overseas^35^. The report concludes that the mean $R_0$ of COVID-19 is approximately 3.28, with a median of 2.79. Compared to the $R_0$ values, the virtual environment closely mimics the COVID-19 situation above the density of 0.01. Therefore, it can be confirmed that the virtual environment can mimic the COVID-19 situation approximately. However, the population density of 0.01 does not spread the disease properly. On the contrary, the population density of 0.04, 0.1, 0.2 excessively spreads the disease. Therefore we conduct our experiment on the population density of 0.02 and 0.03.

**Figure 8:**
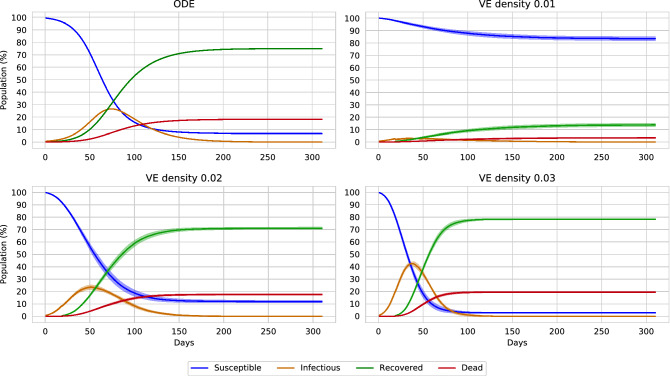
The graph illustrates a comparison of ODE with virtual environment models. The upper left graph shows the ODE variables. The upper right graph shows the virtual environment with a density of 0.01. The lower graphs represent the virtual environment of densities 0.02 and 0.03, respectively. The 0.02 and 0.03 density environments closely relate to the ODE curves.

In comparison to ODE, virtual environments are hardly implemented to study epidemiology. ODE based compartmental models are scientifically accepted, and it is often used to study epidemiology. Therefore, we compare the virtual environment with the ODE model to verify the correctness. However, in the comparison, we omit the exposed state. This is because the RL agent does not apprehend the exposed population data, and the agent is only reported data related to the susceptible, infectious, recovered, and death cases. These circumstances are also illustrated in Fig. 3.

In Fig. 8, we illustrate a comparison of the virtual environment with a general SEIR ODE model. Three virtual environments are reported with a density of 0.01, 0.02, and 0.03. For the ODE model, the implemented equations are conferred below,

5 $$\begin{aligned} \begin{aligned} \frac{dS}{dt}&= -\frac{\beta IS}{N} \\ \frac{dE}{dt}&= \frac{\beta SI}{N} - \alpha E \\ \frac{dI}{dt}&= \alpha E - (\gamma + \mu ) I \\ \frac{dR}{dt}&= \gamma I \\ \frac{dD}{dt}&= \mu I \\\\ Where,&\\&\beta = \text {Transmission rate} = 0.12\\&\alpha = \text {Incubation rate} = 1\\&\gamma = \text {Recovery rate} = \frac{1}{27}\\&\mu = \text {Death rate} = 0.009\\&\text {Reproduction rate, } R_0 = \frac{\beta }{\gamma } = 3.24 \\&N = \text {Total population} = S+E+I+R+D = 10,000 \\&S = \text {Susceptible population} = 9930\\&E = \text {Exposed population} = 0\\&I = \text {Infectious population} = 70\\&R = \text {Recovered population} = 0\\&D = \text {Death} = 0\\ \end{aligned} \end{aligned}$$

The virtual model with a density of 0.01 does not flow the disease due to high spatial distance (and low contact rate) among the population. On the contrary, selecting a density of [0.02, 0.03] produces a similar result to the ODE. For the ODE, the reproduction rate is $R_0=3.24$. For the 0.01 and 0.02 dense virtual environment, the reproduction rate is $3.2\pm 0.30$ and $3.4\pm 0.23$, respectively (reported in Table 1). The graphical flow of the virtual environment reported variables greatly mimics the ODE model. Yet, slight alterations can be noticed due to the value variation of $R_0$. The ODE model illustrates herd immunity^36^, for which around 8% of the population never becomes infected. The minimum portion of infections required to achieve herd immunity can be calculated as $1 - \frac{1}{R_0}$. For the calculated ODE, the minimum value is 88.14% (considering the deaths). Considering the mean $R_0$ of the virtual environments, the 0.02 and 0.03 density environment requires a minimum of 87.75% and 90% population to be infectious. Measuring the flow of the variables and herd immunity, it can be verified that the virtual environment is similar to ODE models.

### Agent comparison

The memory-based agent is trained in the virtual environment with random initialization of infections. A scale of [1, 20]% infectious population is randomly initialized for each virtual environment play/episode. Also, there is no fixed day limit when the disease of the virtual environment fully mitigates. Therefore, each episode is kept running until the disease fully mitigates (zero active cases and exposed state). We initially trained the agent with a 30 days memory, and it took nearly 10 days to complete the training of 7000 episodes. Apart from the 30-day memory agent, we further trained agents with different memory lengths, including 7, 15, 45, and 60 days. Each of the agents is named based on the memory length (i.e., M45 refers to the agent with 45 days of memory). However, to reduce the computational complexity, we initialized the M7, M15, M45, and M60 models with the pre-trained weights of the M30 model.

**Figure 9:**
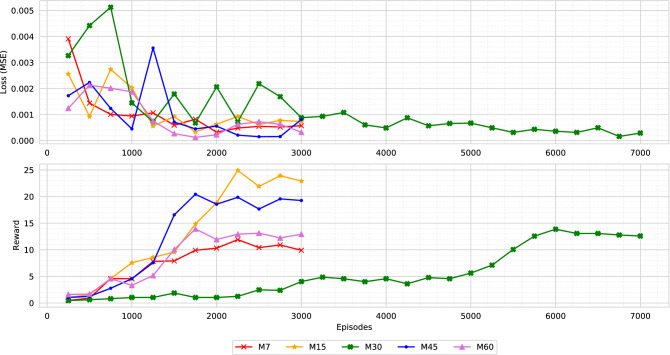
The figure illustrates the loss (upper) and reward (lower) comparison of the agents while training. Each of the agents was evaluated after 250 episodes. A single evaluation is presented as a mean of ten runs, and it is guaranteed that every model is tested on the same environment scenario. The M30 agent was trained for 7000 episodes. The M7, M15, M45, and M60 agents were initialized with the M30 agent’s trained weights. Therefore, these agents converged to an optimal state within 3000 episodes.

**Figure 10:**
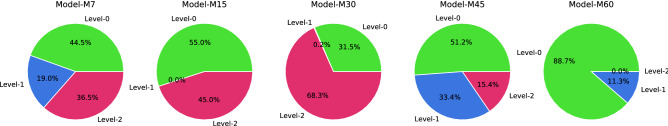
The figure represents the ratio of the actions performed by each agent. Agent M15 and M30 mostly instruct level-0 and level-2 restrictions. Agent M7 and M45 charge all types of rules. Whereas, agent M60 mainly engage level-0 and level-1 restrictions.

**Figure 11:**
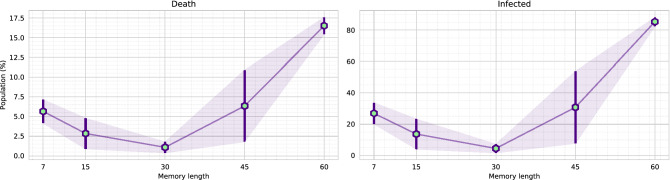
The graphs represent the average deaths and infections caused while an agent performed actions. The results are presented in mean value with standard deviation error. It is also guaranteed that every model is tested in the same environment scenario.

**Figure 12:**
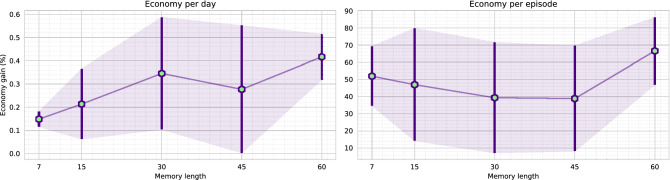
The graphs depict the economic benefits secured while the agent performed. The left graph presents an average economy gain per day, and the right illustrates the total economic gain per episode. The difference between average and total gain is due to the dynamic day of the environment. The environment is kept running until the disease fully mitigates. Therefore, agents that require a higher time to mitigate a disease gets higher time to make an economic profit.

**Figure 13:**
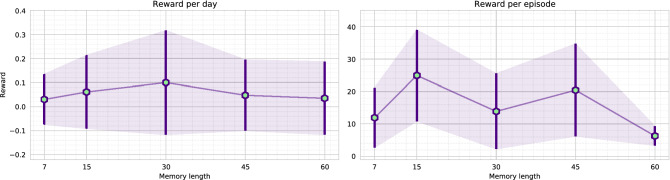
The left graph exhibits the agent’s reward points on average (per day), and the right illustrates total economic gain for an episode. It is guaranteed that the comparisons are executed in the same environmental conditions.

**Figure 14:**
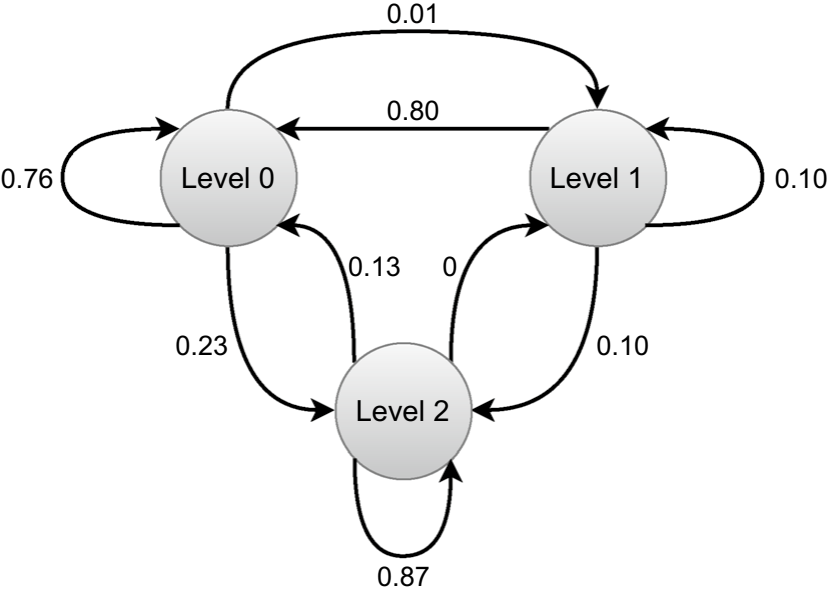
The figure illustrates the transition probability of agent M30. Each node represents the asserted regulation. The agent recommends level-2 restrictions instead of level-0 and level-1 restrictions. The agent almost ignores level-1 regulations in the decision process.

Figure 9 presents a comparison of loss and reward values. As the figure depicts, M30 model requires around 6000 episodes to achieve a better reward. Whereas, after some fluctuation in the loss value, the other pre-initialized models (M7, M15, M45, M60) converge to optimum reward within 3000 episodes. The graph concludes that agent M15 produces a higher score per-play, followed by agent M45 and agent M30. As the reward function (explained in Eq. 1) is formulated by aggregating the target objectives (death, economy, and active cases), we imply that an agent is optimal if it achieves a higher aggregated reward. Therefore, the agent M7 and M15 reaches the optimal state on epoch 2250. In comparison, agent M45 and M60 reach optimal state comparatively earlier, on epoch 1750. Moreover, agent M30 reaches an optimal state on epoch 6000. We further investigate for an optimal agent that not only secures higher rewards but also reduces the disease infection and gains better economic profit. Further comparisons are made with the weights for which each of the agents acquired maximum reward (illustrated in Fig. 9). Also, the environment scenario used to evaluate the agents are the same for all the evaluations (Figs. 9, 10, 11, 12, 13, 14).

Figure 10 represents an investigation on the actions performed by the agents. The best performing agent M15, mostly instructs level-0 and level-2 restrictions. Whereas, the second-best agent M45 provides all types of rules. The third-best agent M30 mainly provides level-0 and level-2 regulations. Figure 11 illustrates the death and infections that occurred due to the agents’ execution. Agent M30 achieves minimal infections and deaths due to the strict lockdown policy. In contrast, agent M15 and agent M45 place second and third, in the comparison, respectively.

Further, Fig. 12 illustrates a different scenario of the economic situation of the environment. We demonstrate the financial gains in two different aspects, per-day economy gain (total economy divided by the number of actions/days) and the entire economy gain per episode. Although agent M30 mostly places level-2 restrictions, it achieves better per-day financial profits than agent M15 and M45. In contrast, agent M15 gains better economic profit from each episode. Agent M15 performs more extended actions and keeps the disease propagating for a longer time. For longer runs, agent M15 receives a better cumulative reward than most of the agents.

Moreover, we validate the assumption by performing a close re-investigating the agents’ best rewards. Figure 13 illustrates the scenario. The graph validates that the agent M30 receives a better on-average reward for each day. As the agent M30 also mitigates the disease faster, it receives better economic advantages than any other model. Overall, we can conclude that the agent M30 poses some advantages such as,

- The agent M30 quickly mitigates the disease.
- It ensures a minimal spread of the disease. The minimal spread of disease also causes lesser death.
- It also achieves a better economic balance comparing to any other agent. Comparatively better than agent M15 and M45 agents’ average economy. The agent quickly mitigates the disease, and it does not need to provide lockdowns in the future. Therefore, it has better economic advantages.

**Table 2:** The table accumulates the overall comparisons of the memory-based agents.

	M7	M15	M30	M45	M60
Points (per day)	5	2	**1**	3	4
Economy (per day)	5	4	2	3	**1**
Infected	3	2	**1**	4	5

For each comparison, we initialize rankings for each agent. Lower orders are better. Agent M30 comparatively performs better than the rest of the agents.

Minimum orders are marked as bold.

Table 2 further aggregates the findings of the overall agent comparisons. It further validates that the agent M30 performs optimal, and it also guarantees minimal infection with higher economic benefits. Agent M30 has some architectural benefits compared to the other agents. The virtual environment is initialized so that a cycle of disease’s propagation can be approximately discovered in 30 days. Therefore, agent M30 formulates a batter perception because it can receive the full result in about 30 days. Consequently, due to this advantage, we assume that agent M30 can optimally mark the optimum global position of the dimensions generated by the environment. On the contrary, agent M15 could not target the disease’s propagation cycle and greedily converges to a state where it can achieve higher rewards by keeping the environment active for a longer time. Although agent M45 and M60 receive the same scenario as agent M30, they fail to establish appropriate reasoning to apply restrictions. Agent M45 and M60 can not appropriately target disease propagation from the input sequences due to increased features. As a result, agent M45 also converges to a state where it can gain better rewards in total. In Fig. 14, the transition probability of the agent M30 is presented. As illustrated in Fig. 10, the agent does not apply level-1 restrictions and mostly performs level-2 regulations. Yet, it achieves a comparatively better economic balance. In the next section, we illustrate the decisions that the agent M30 takes to mitigate the disease.

**Figure 15:**
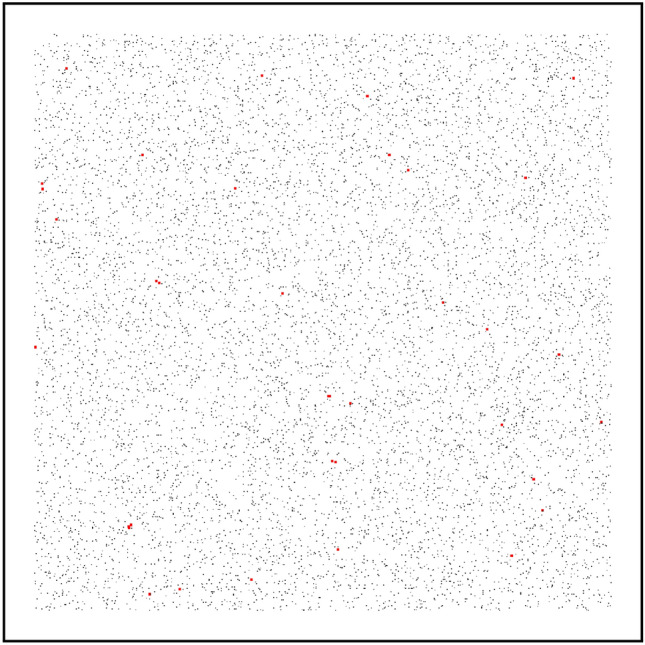
This graph represents the initial state of the pandemic. The black dots denote the position of the susceptible population. The red dots denote the position of the infectious population. The virtual environment contains 10,000 population, in which, 70 (0.7%) of them are infectious. This is a challenging scenario because the infectious is heavily spread all over the regions. The density of the environment is set to be 0.02.

### Evaluation of different control sequences

**Figure 16:**
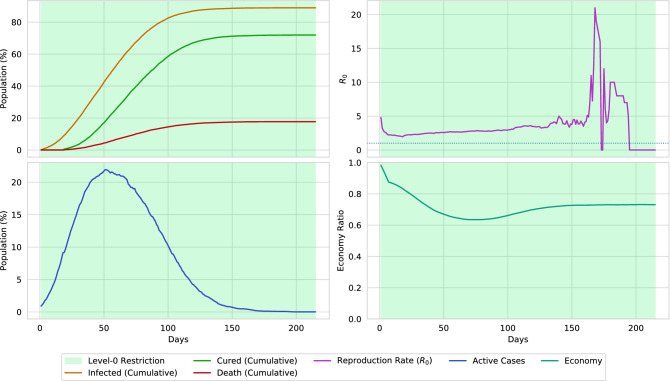
A simulation of the virtual environment (0.02 population density) by placing level-0 restriction. The upper-left portion illustrates the cumulative sum (in percentage) of infected, cured, and dead of the overall population. The upper-right portion illustrates the reproduction rate of the disease. The lower-left portion indicates the percentage of the active cases of the population. The lower-right portion determines the economical state through the spread of the disease. A massive surge of active cases is reported on reaching the 50th day of the pandemic. Around 20% of the population dies due to the disease if no lockdown is placed and no social-distancing is maintained.

Figure 16 presents a datasheet of the virtual environment simulation and Fig. 15 represents the initial positioning of the infectious population over the environment. The datasheet is separated into four individual graphs. In the current simulation, no lockdown is placed (level-0 restriction). The graph indicates a raise in active cases by simultaneously infecting 20% of the population. Without placing any lockdown, the disease affects more than 80%, among which, around 20% of the population loses their lives. Due to the huge decrease in the population, an impact is also measured in the economical state of the environment. As the non-survivals could not contribute to the economy, the economic ratio of the environment falls around 0.20 due to the loss of the population. Therefore, considering the economy, it can be determined that placing no lockdowns in a pandemic situation may not be a good solution. The reproduction rate of the disease is mostly in a close interval of 2–5. However, a surge in the reproduction rate is reported after passing 160 days of the pandemic, due to the superspreaders.

**Figure 17:**
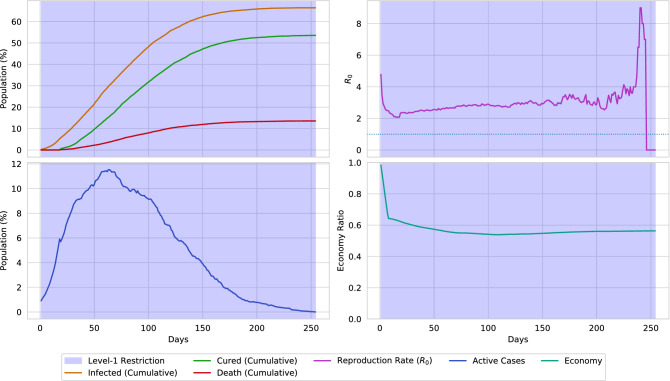
A simulation of the virtual environment (0.02 population density) only if social-distancing is maintained. Due to social-distancing, the spread of the disease is reduced. Hence, the total number of infections is reduced by 20%, along with a reduction in deaths by 10%. Although the economical ratio is reduced by 0.2, it is considerate, relating to the reduced spread of the virus.

**Figure 18:**
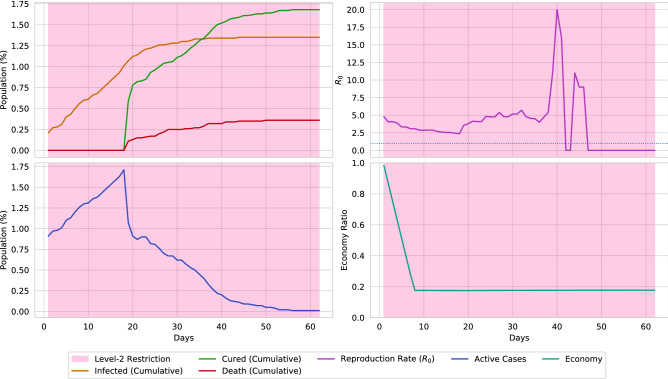
A simulation of the virtual environment if full lockdown is ordered. Due to strict lockdown, the spread of the virus fully stops after 60 days. However, this is almost impossible to occur in a real-world scenario. Furthermore, lockdown causes the economical ratio to be decreased to less than 0.2.

The effect of social-distancing (level-1 restriction) is presented in Fig. 17. By maintaining social-distancing, around 20% spread of the disease can be reduced, along with 10% fewer deaths. Also, the surge of active cases is reduced by around 10%. However, due to social-distancing, the economic ratio is decreased by around 0.2. The impact of lockdown (level-2 restriction) is presented in Fig. 18. From the illustration, it can be stated that placing lockdown heavily decreases the spread of disease. On the contrary, placing lockdown also causes the economy to collapse. The simulation also points out that the spread of disease can be fully halted by placing a 63 days lockdown. However, in the real world scenario, complete elimination of a disease through lockdown is near impossible.

**Figure 19:**
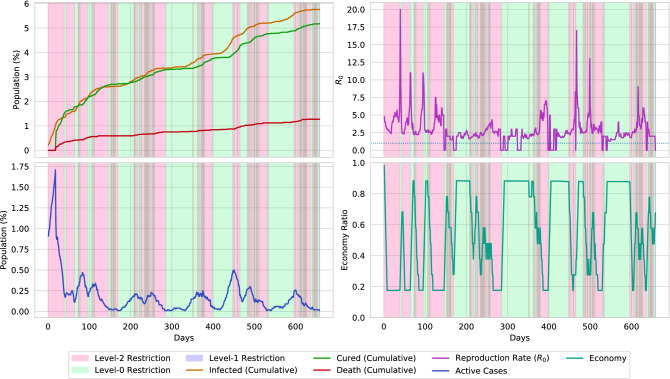
The graphs represent the movement restrictions provided by the agent. The red region of the graph denotes the days when a lockdown is placed. The green region of the graph denotes the days when no lockdown is placed. In the early stage of the environment, the agent places multiple 20–40 days lockdown to reduce the spread of the disease. In the later stage, to control the resurgence of the disease, the agent performs a cyclic lockdown (1–3 days cycle) followed by a 10–15 days lockdown to reduce the future spread of the virus. It can be also analyzed that the agent mostly follows this pattern when both the active cases percentage and the reproduction rate is high.

**Figure 20:**
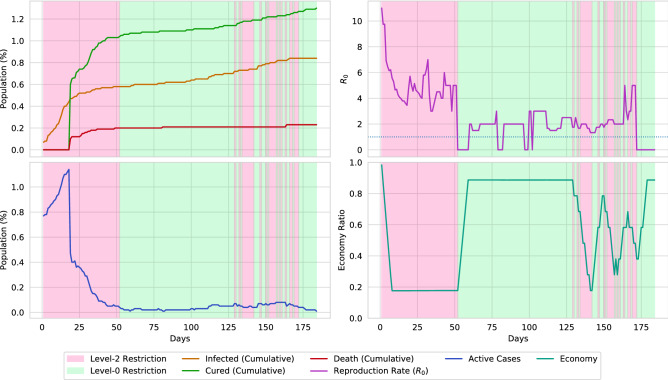
This graph illustrates the actions performed by the agent in a 0.01 population density environment. The other environmental parameters are kept unchanged. The graph resembles a similar action pattern of the agent observed in a 0.02 population density environment. However, less population is infected due to the lesser spread of the disease.

**Figure 21:**
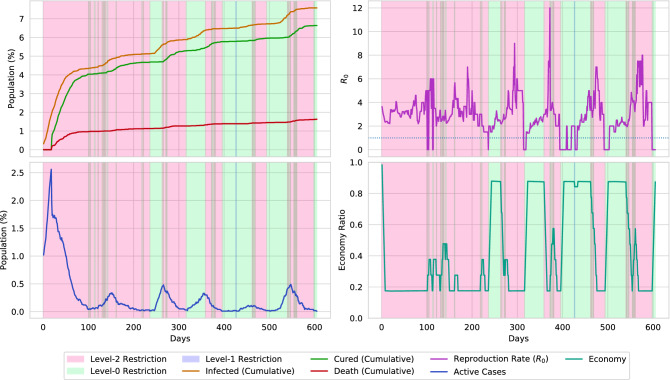
This graph illustrates the actions performed by the agent in a 0.03 population density environment. The other environmental parameters are kept unchanged. The graph resembles a similar action pattern of the agent observed in a 0.02 population density environment. However, due to increased population density, the spread of disease is also increased. Therefore, the agent mostly places strict lockdown instead of cyclic lockdown.

Figure 19 illustrates the restrictions that the agent placed in the virtual environment of population density 0.02. The initial state of the environment starts with a devastating pandemic situation, in which, the disease infects almost 1% of the population. Therefore, the agent places multiple 30–40 days of lockdown segments to reduce the spread of the disease. Then the agent removes the restrictions and stables the economy. However, multiple smaller peaks of active cases are reported in an approximately 100 days cycle. The agent reduces the spread of the disease by performing two types of actions. At first, the agent activates a cyclic lockdown to level the spread of the virus by keeping the economy steady as much as possible. Finally, the cyclic lockdown is followed by a 10–20 days long lockdown. By further analyzing the reproduction rates of the environment, it can be concluded that this combination optimally reduces the reproduction rate below 1. Reducing the reproduction rate causes the spread of the disease to be halted. In Figs. 20 and 21, the action sequences of the agent are illustrated for an environment of population density 0.01 and 0.03, respectively. In both cases, the agent follows a cyclic lockdown if the situation is less severe; otherwise, it places a full lockdown. Furthermore, by closely evaluating the reproduction rate and active cases of the environment, a pattern of the lockdown placement can be observed.

The agent places lockdown based on the active cases and the reproduction rate. However, it can be observed that the agent sometimes avoids placing lockdown when the reproduction rate is high. The agent only places lockdown when the value of active cases and reproduction rates are high. It further removes the lockdown when the reproduction rate is less than 1. To discover the reason for the action, let us consider the following formula,

6 $$\begin{aligned} \delta Inrease_{disease} = ActiveCases \times R_0 \end{aligned}$$

The equation formulates the possible number of people who may get infected in the next day. The reproduction rate $R_0$ represents the average number of newly infected cases caused by an infectious person, and the value of *ActiveCases* indirectly represents the number of infected persons in a single day. Therefore, the increase in infectious cases can generally be formulated using Eq. (6). The agent places strict lockdown actions when the value of Eq. (6) becomes too high. On the contrary, for minor cases, the agent follows a cyclic lockdown phase. This causes optimally controlling the spread of the disease below a particular percentage.

**Figure 22:**
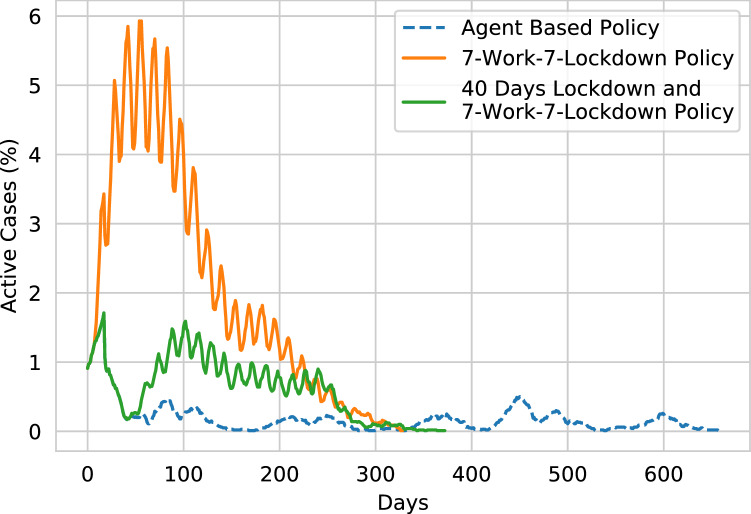
The graph presents a comparison of the agent’s policy with the traditional n-work-m-lockdown policy. The comparison is formed on a 10,000 population with a density of 0.02. By only maintaining a 7-work-7-lockdown policy, a rapid spread of the virus can not be halted, and therefore, a total of 34.5% of the population gets infected. Furthermore, if the 7-work-7-lockdown policy is applied after a full lockdown of 40 days, the overall infection is decreased to 11.5%. However, the agent generated policy mostly flattens the curve.

In Fig. 22, we further compare the agent’s policy with the traditional n-work-m-lockdown policy. From the comparison, it can be justified that only maintaining the n-work-m-lockdown policy is not an optimal solution to mitigate a pandemic. Furthermore, adding 40 days of full lockdown before following the n-work-m-lockdown policy reduces the first surge of the disease. However, the n-work-m-lockdown policy does not control the spread of the disease properly. Therefore, a resurgence of the disease is observed. From the general comparison, it can be validated that an agent can optimally control a pandemic crisis if proper training method is implemented.

## Discussion

The paper motivates the readers towards the achievements and advancements of reinforcement learning through its application for controlling the pandemic crisis. We introduce a virtual environment that mostly relates to a pandemic situation, and sedulously investigate new tactics to mitigate disease by applying reinforcement learning. In what follows, we perform a pensive analysis of the impact of lockdown, social-distancing, and using agent-based solutions to prevent the mitigation of disease. We find our proposed scheme to be convincing in achieving optimal decision balancing the overweening pandemic and economic situation. We strongly believe that the contribution of this research endeavor will unite the epidemic study with reinforcement learning, and may help the human race to defend against the pandemic crisis.

## Supplementary information

Supplementary Information 1
