# Supplementary material for: Exploring optimal control of epidemic spread using reinforcement learning
Source: Sci Rep. 2020 Dec 16;10:22106. doi: 10.1038/s41598-020-79147-8 (PMC7744528; doi:10.1038/s41598-020-79147-8)
Supplement: Supplementary file 1 — Supplementary Information 1 [file 41598_2020_79147_MOESM1_ESM.pdf]

Algorithm 1: A pseudocode of the execution process of the virtual environment.

Input: The size of the grid s,
The size of population N,
Number of infectious population M,
Number of days staying exposed E_t,
Number of days staying infectious I_t,
Number of daily movements M_t,
Number of days D

```
S ← {(x, y, d) ∈ ℕ|0 ≤ x, y ≤ s and d = 0}  and  |S| = N − M ;   /* susceptible population */
E ← {} ;                                                   /* exposed population */
I ← {(x, y, d) ∈ ℕ|0 ≤ x, y ≤ s and d = 0}  and  |I| = M ;   /* infectious population */
R ← {} ;                                                   /* recovered population */
P ← S ∪ E ∪ I ∪ R ;                                        /* total population */
Economy ← 0 ;                                              /* total economic transaction */
/* loop for each day */
for day ← 1 to D do
    /* loop for each step */
    for m_t ← 1 to M_t do
        /* loop for each person */
        for p ∈ P do
            /* ℤ ∩ [−1, 1] defines picking a random integer from -1, 0, and 1 */
            x_t ← max(min(p(x) + ℤ ∩ [−1, 1], s), 0) ;   /* making valid movements in the grid */
            y_t ← max(min(p(y) + ℤ ∩ [−1, 1], s), 0);
            z_t ← p(z) + 1 ;                              /* updating the day counter */
            /* if the person is in recovered state */
            if p ∈ R then
                P ← (P − p) ∪ {(x_t, y_t, 0)} ;           /* no state upates for recovered population */
            /* if the person is in infectious state */
            else if p ∈ I then
                /* ℕ ∩ [0, 6] defines picking a random integer in range [0, 6] */
                if z_n − ℕ ∩ [0, 6] ≥ I_t then
                    /* randomly choose if a person survives, and the probability distribution of choosing 1
                       over 0 is 1:4 */
                    if ℕ ∩ [0, 1] = 1 then
                        P ← P − p ;                       /* dead person are removed from the states */
                    else
                        R ← R ∪ {(x_t, y_t, 0)} ;         /* recovered person is moved to recovered state */
                        P ← (P − p) ∪ {(x_t, y_t, 0)};
                    I ← I − p;
                else
                    P ← (P − p) ∪ {(x_t, y_t, z_t)};
                    I ← (I − p) ∪ {(x_t, y_t, z_t)};
            /* if the person is in exposed state */
            else if p ∈ E then
                /* ℕ ∩ [0, 1] defines picking a random integer 0 or 1 */
                if z_n − ℕ ∩ [0, 1] ≥ E_t then
                    E ← E − p;
                    I ← I ∩ {(x_t, y_t, 0)};
                    P ← (P − p) ∪ {(x_t, y_t, 0)};
                else
                    E ← (E − p) ∪ {(x_t, y_t, z_t)};
                    P ← (P − p) ∪ {(x_t, y_t, z_t)};
            /* if the person is in susceptible state */
            else
                /* check if the person is in close contact with any of the infectious person */
                if (x_n + [−1, 1], y_n + [−1, 1], ℕ) ∈ I then
                    I ← I ∪ {x_t, y_y, 0} ;               /* move the person in exposed state */
                    P ← (P − p) ∪ {(x_t, y_t, 0)};
                else
                    P ← (P − p) ∪ {(x_t, y_t, z_t)}
            /* except of the person is not infectous (and not dead) he/she contributes to the economy */
            if p ∉ I then
                Economy ← Economy + ℝ ∩ [0.8, 1];
```
